# Spouted Bed Drying of Fresh Papaya Seeds (*Carica papaya* L.): Hydrodynamic Behavior and Statistical Analysis

**DOI:** 10.1021/acsomega.6c01856

**Published:** 2026-05-28

**Authors:** Alcino Matos de Azevedo Pontes Neto, Dhessyca Maria Vitoria Gonçalves Soares, Marlice Cruz Martelli, Davi do Socorro Barros Brasil

**Affiliations:** Graduate Program in Chemical Engineering, Institute of Technology, 37871 Federal University of Pará, 66075-110 Belém, Brazil

## Abstract

The processing of papaya (*Carica papaya* L.) generates substantial volumes of bioactive-rich seed residues that require efficient drying to prevent degradation. However, the hydrodynamic behavior of fresh seeds, characterized by high surface moisture and mucilaginous adhesion, remains poorly documented under spouted bed conditions. This study systematically investigated the drying performance of papaya seeds using a Box–Behnken experimental design. Initial trials showed that removing the sarcotesta drastically reduced the minimum spouting velocity (*u*_*ms*_) from 1.94 to 0.98 ms^–1^ and the peak pressure dropped from 1200 to 490 Pa, highlighting the dominant role of surface cohesion. A 15 min flash-drying pretreatment at 4 m^3^ min^–1^ was essential to reduce surface stickiness and enable stable spouting. The experimental design evaluated air temperature (40–60 °C), processing time (20–60 min), and airflow rate (1.0–2.0 m^3^ min^–1^). Quadratic regression models for moisture content and water activity (*a*_*w*_) exhibited excellent fit (*R* ^2^ > 0.99; *R*_*adj*_^2^ > 0.97) with no significant lack of fit (*p* > 0.05). Multiresponse optimization via desirability profile (≈60 °C, ≈60 min, and 1.61 m^3^ min^–1^) converged with experimental results (*U* = 7.2% and *a*_*w*_ = 0.57), reaching the thermodynamic safety threshold (*a*_*w*_ < 0.60) for microbial inhibition. These findings demonstrate the potential of spouted bed technology for the scalable and sustainable valorization of sticky agro-industrial residues, ensuring material stability for future bioactive compound extraction.

## Graphical abstract

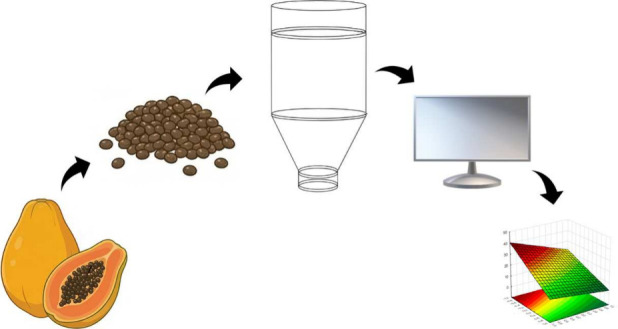

## Introduction

The rapid expansion of the global fruit processing sector has intensified the generation of agro-industrial residues, which now represent hundreds of millions of tons of biomass annually. Despite their abundance, these byproducts remain underutilized, even though they contain structurally diverse bioactive compounds and platform molecules of technological interest.^1,2^ Within this scenario, papaya (*Carica papaya* L.) emerges as a relevant tropical crop whose processing chain produces substantial quantities of peels and seeds, materials frequently discarded despite their documented phytochemical richness. The sustainable management and valorization of such residues are directly aligned with Sustainable Development Goal 12 (Responsible Consumption and Production) established by the United Nations within the 2030 Agenda for Sustainable Development.^3^

Papaya seeds, specifically, harbor vast biotechnological and nutritional potential. Recent studies demonstrate that these seeds are rich sources of bioactive compounds, high-quality oils with high oleic acid content, and mucilage enriched with homogalacturonan domains.^4–6^ Furthermore, the harvest time and storage period directly influence the germination, vigor, and overall physiological quality of the material.^7,8^ For these high-value-added bioproducts to be extracted or for the seeds to maintain their commercial viability, efficient moisture removal becomes an indispensable preliminary step.

Drying acts as a fundamental unit operation to extend the shelf life and ensure the physicochemical stability of biological materials.^9^ However, handling coarse-grained particles presents considerable hydrodynamic challenges. Because of their diameter, porosity, and specifically the presence of a highly cohesive mucilaginous layer (the sarcotesta), papaya seeds exhibit strong interparticle adhesion. This characteristic frequently places them in particle categories that do not fluidize properly in conventional systems, tending toward air channeling or the formation of unstable bubbles.^10^ This behavior demands the use of technologies that promote a more rigorous and uniform gas–solid contact.

To overcome the limitations of classical fluidization in the treatment of coarse and cohesive particles, the spouted bed technique was developed and consolidated in chemical engineering.^11–13^ The spouted bed promotes a cyclic movement of particles through a high-velocity central region and a descending annular region, ensuring highly efficient heat and mass transfer rates, which is ideal for preventing the overheating of thermosensitive seeds.^14^ While continuous advancements often rely on the insertion of internal devices like draft tubes and fountain deflectors to improve stability,^15–18^ these accessories can complicate the operation with sticky materials. Unlike standard spouted beds, the equipment utilized in this study features a modified conical-cylindrical geometry with an atypically large air inlet diameter (9.47 cm). This specific design preserves the heat and mass transfer benefits of the conical section but significantly reduces the pressure drop required to achieve a stable spouting state while promoting a lower, controlled fountain height, eliminating the need for internal draft tubes or deflectors. The system is also sealed with a fine mesh screen and coupled to a cyclone to ensure the recovery of elutriated fines.

Despite its notable advantages, operating a spouted bed involves a complex interaction between hydrodynamic and operational variables, and the modeling and optimization of these parameters require robust mathematical approaches. The use of design of experiments (DoE) tools allows for the rigorous investigation of the effects of multiple independent variables and their simultaneous interactions in a structured manner.^19^ This statistical approach is crucial for mapping response surfaces and determining the optimal operating conditions that maximize the process efficiency.

The literature still lacks comprehensive studies on how surface structures, such as the sarcotesta, control fluid dynamic stability. This study makes two original contributions: First, it elucidates how the presence or absence of the sarcotesta directly influences the hydrodynamic behavior of the bed, and second, it demonstrates the effectiveness of a modified spouted-bed geometry with an enlarged air inlet. This geometry overcomes the inherent cohesiveness of these seeds without the need for internal devices. By applying Box–Behnken experimental designs, this work also models and optimizes the operating conditions. The results provide essential fluid dynamic and stabilization data for engineering projects and scaling up.

## Results and Discussion

### Physical Characterization

Papaya seeds (*Carica papaya* L.) were characterized regarding their physical properties, with *n* = 80 for mean diameter and sphericity and *n* = 5 for density, with the results described in Table 1.

**Table 1:** Physical Properties of Papaya Seeds under Different Processing Conditions^a^

**Physical Property**	**Sample Condition**	**Experimental Value**
Mean Diameter, *d*_*p*_	With sarcotesta	4.08 ± 0.07 mm
	Without sarcotesta	3.21 ± 0.04 mm
	Dried	3.42 ± 0.10 mm
Sphericity, ϕ	With sarcotesta	0.84 ± 0.02
	Without sarcotesta	0.79 ± 0.02
	Dried	0.88 ± 0.01
Particle Density, ρ_*p*_	With sarcotesta	1093 kg m^–3^
	Without sarcotesta	672 kg m^–3^
	Dried	978 kg m^–3^

a Values represent the mean ± standard deviation.

The mean particle diameter (*d*_*p*_) exhibited significant variation depending on the seed condition, decreasing from 4.08 mm (fresh seeds with sarcotesta) to 3.42 mm after the drying process. This reduction is a direct consequence of moisture loss and subsequent structural collapse of the outer layers. In drying operations, volume shrinkage is a characteristic phenomenon in particulate biological materials as moisture evaporates.^9^ The even smaller diameter of 3.21 mm observed for seeds manually stripped of their sarcotesta confirms that this mucilaginous envelope represents a substantial fraction of the fresh seed’s volume.

Concurrently, the sphericity (ϕ) increased from 0.84 in fresh seeds to 0.88 in the dried samples. This morphologic rounding can be attributed to the continuous interparticle friction inside the spouted bed and the uniform shrinkage of the sarcotesta around the exotesta during the process.^9^

Furthermore, the particle density (ρ_*p*_) varied markedly, dropping from 1093 kg m^–3^ in the fresh state to 978 kg m^–3^ after drying. Variations in specific weight and density are critical physiological and physical markers for papaya seeds, often correlating with their maturation and structural integrity.^8^ This indicates that the mass loss due to water evaporation was proportionally greater than the volume shrinkage of the seed.

On the basis of the measured initial diameter and density, the fresh papaya seeds are strictly classified within Geldart’s Group D.^10^ Particles in this group are characterized by being coarse and dense, typically presenting severe fluidization issues in conventional fluidized beds such as slugging and severe air channeling. Therefore, the implementation of a spouted bed is fully justified, as its central jet and annular downward flow provide the vigorous cyclic motion necessary to ensure homogeneous gas–solid contact and efficient heat transfer for this specific class of biomaterials.^12,14^

### Seed Fluid Dynamics

The fluid dynamic behavior of seeds with intact sarcotesta (Figure 1, which represents the mean profile of three independent experimental runs) exhibited severe hysteresis and erratic pressure fluctuations. Instead of a sharp transition to a stable spouting regime, the bed presented an unstable intermediate pressure region without immediate stabilization. This atypical behavior is primarily attributed to the strong cohesive and adhesive forces exerted by the moisture-rich mucilage, which promotes particle agglomeration. In this case, *ΔP*_stable_ fluctuated between 1120 and 1340 Pa across the experimental replicates (averaging approximately 1200 Pa) just to break the initial packing. The consistent recording of this high-pressure variation range across independent runs confirms the reproducibility of these erratic patterns for mucilage-coated seeds.

**Figure 1:**
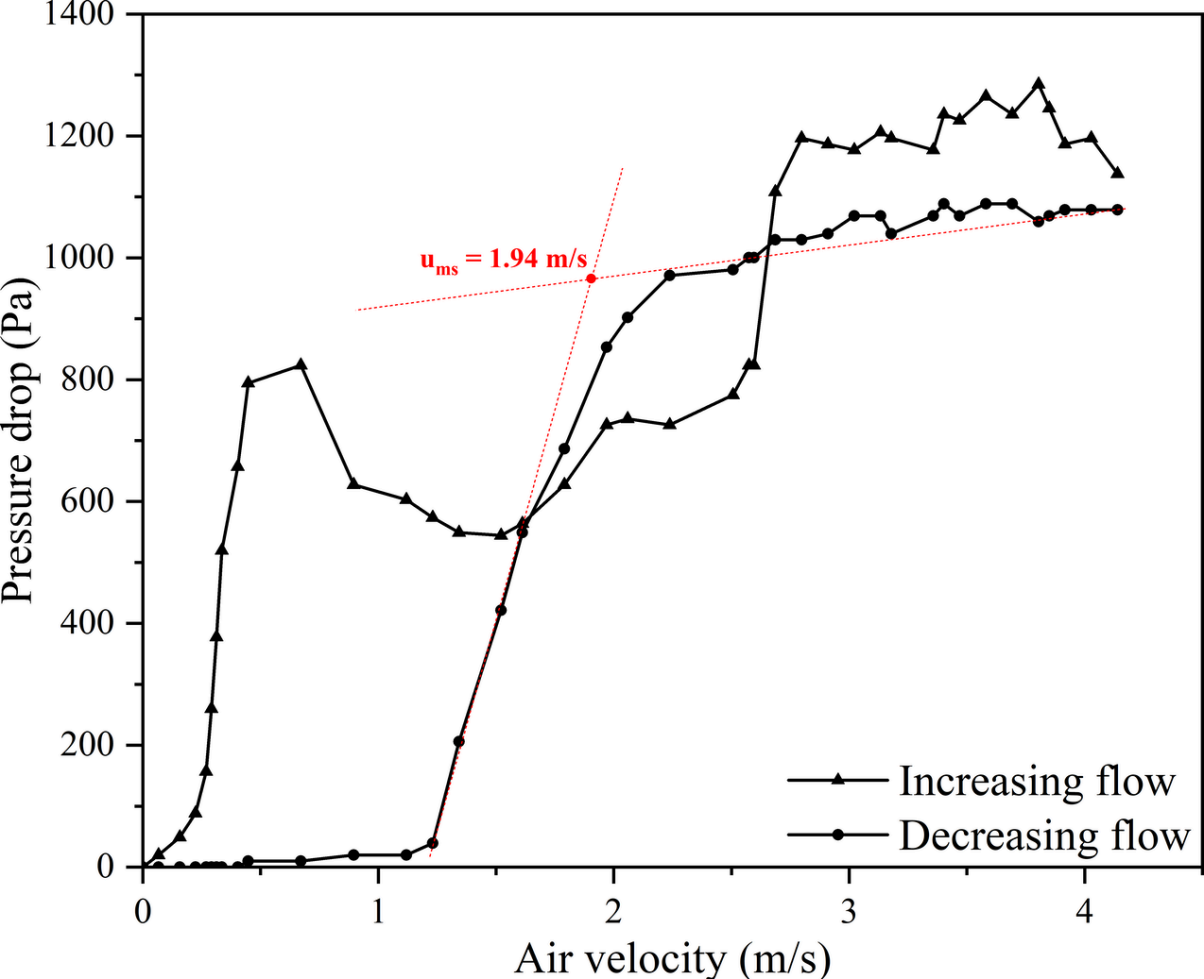
Fluid dynamic pressure drop (*ΔP*) versus air velocity for papaya seeds with intact sarcotesta. The red dashed lines indicate the graphical determination of the minimum spouting velocity (*u*_*ms*_).

Conversely, the mechanical removal of the sarcotesta drastically altered the bed’s hydrodynamics (Figure 2, which also represents the mean profile of the experimental triplicates). The system exhibited a classic spouted bed pressure drop curve, characterized by a distinct peak pressure drop followed by a sharp decline to a stable operational pressure drop (*ΔP*_stable_) that consistently remained between 470 and 520 Pa (averaging approximately 490 Pa). The minimum spouting velocity (*u*_*ms*_) was also significantly reduced, confirming that removing the mucilaginous layer eliminates cohesive restrictions, thereby facilitating the stable, cyclic gas–solid motion that defines the regular spouting regime.^18^ The narrow pressure range and the minimal variance observed across the independent repetitions for these clean seeds highlight a highly reproducible and stable fluid dynamic behavior, as summarized in Table 2.

**Figure 2:**
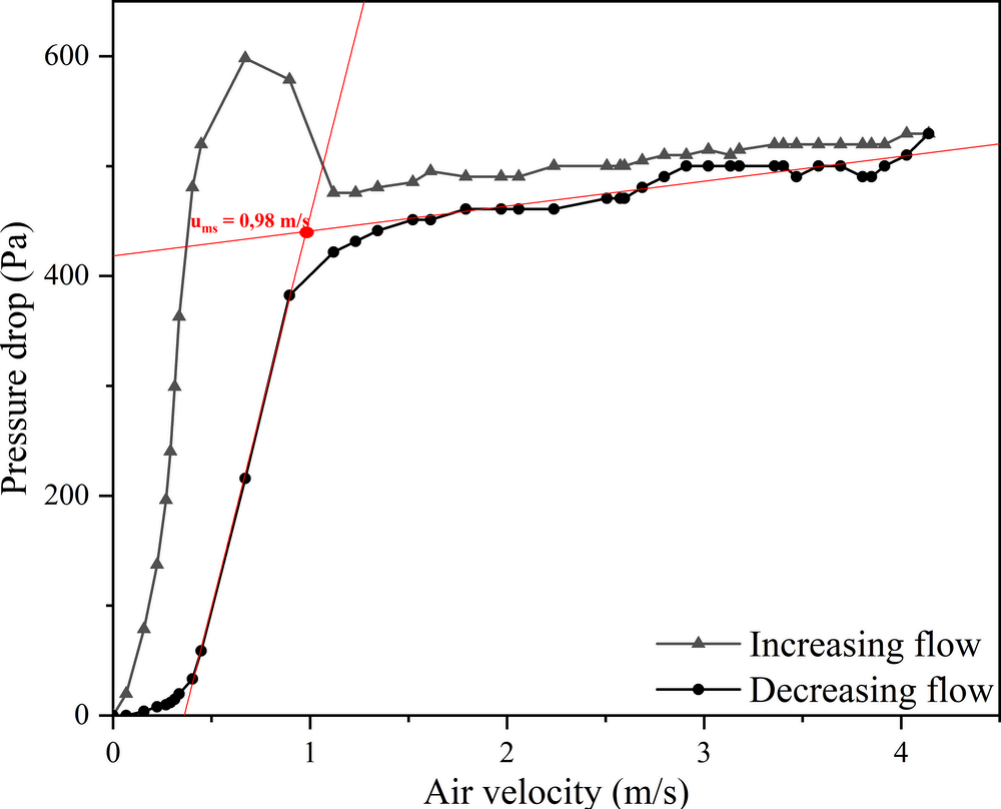
Fluid dynamic pressure drop (*ΔP*) versus air velocity for papaya seeds without sarcotesta. The red dashed lines indicate the graphical determination of the minimum spouting velocity (*u*_*ms*_).

**Table 2:** Fluid Dynamic Parameters of Papaya Seeds under Different Physical Conditions^a^

**Seed Condition**	***u***_***ms***_ **(ms^–1^)**	**Δ*P*_stable_ (Pa)**
With sarcotesta	1.94 ± 0.03	1230 ± 110
Without sarcotesta	0.98 ± 0.05	495 ± 25

a Values represent the mean ± standard deviation.

When comparing the experimental data with classical fluid dynamic models (Table 3), the Ergun equation provided a closer velocity estimation for seeds with sarcotesta (relative error of 20.46%) than the Mathur–Gishler model. This occurs because the highly cohesive bed acts more like a static packed bed with localized channeling rather than a true spouted bed. However, for seeds without a sarcotesta, the Mathur–Gishler model demonstrated a much better fit (error of 16.02%). Given the empirical nature of this correlation, this margin of error is highly acceptable and analytically confirms the re-establishment of the classical spouting regime^11^ once the sticky outer layer is removed.

**Table 3:** Validation of Theoretical Models for Predicting the Minimum Spouting Velocity (*u*_*ms*_) under Different Seed Conditions

**Seed Condition**	**Method**	***u***_***ms***_ **(ms^–1^)**	**Relative Error (%)**
With sarcotesta	Experimental	1.94 ± 0.03	
	Ergun Equation	2.34	20.5
	Mathur–Gishler	0.92	52.4
Without sarcotesta	Experimental	0.98 ± 0.05	
	Ergun Equation	0.57	42.0
	Mathur–Gishler	0.82	16.0

The divergence observed among our experimental values, theoretical predictions, and recent literature also suggests a sensitivity of the *u*_*ms*_ parameter to the equipment’s base geometry and nozzle design, a dependency that has been demonstrated in recent numerical and experimental studies.^17^ For instance, while this study achieved stable spouting at *u*_*ms*_ = 0.98 ± 0.05 ms^–1^ for dried seeds, Reis et al.,^16^ operating with similar biomass in a standard spouted bed (*D*_*c*_ = 0.25 m and *D*_*i*_ = 0.05 m), reported fluidization velocities exceeding 13 ms^–1^. It is hypothesized that this order-of-magnitude discrepancy is primarily driven by the inlet flow area of this custom equipment. A significantly larger nozzle diameter (9.74 cm) expands the cross-sectional area at the base, drastically reducing the superficial gas velocity required to balance the gravitational and drag forces acting on the Geldart Group D particles. Consequently, this design allows for stable and energy-efficient operation at flow regimes considerably lower than those predicted by standard correlations.

### Evaluation of the Drying Process

Before evaluating the process variables via the Box–Behnken design, the initial handling of the biomaterial must be addressed. The drying study was conducted using a heterogeneous batch of papaya seeds regarding the presence of sarcotesta. To ensure consistent fluidization and overcome the strong interparticle cohesive forces of the fresh mucilage previously discussed, a flash drying pretreatment was systematically applied to the bed.

This pretreatment consisted of subjecting the seeds to an initial air jet at the equipment’s maximum flow capacity (4 m^3^ min^–1^) for 15 min. This brief but intense pneumatic intervention was highly effective, as the initial moisture content rapidly dropped from approximately 78% to 66%, significantly mitigating the critical cohesion of the bed. Furthermore, the high fluid dynamic stress and rapid superficial evaporation during this stage caused the sarcotesta of a substantial fraction of the seeds to burst. This functional breakdown of the mucilaginous envelope practically replicated the hydrodynamic benefits of mechanical sarcotesta removal, establishing a stable spouting regime and allowing for consistent cyclic motion during the subsequent statistically designed experimental runs.

The experimental matrix generated by the Box–Behnken design (BBD), alongside the corresponding responses for final moisture content (*U*, % w.b.) and water activity (*a*_*w*_), is presented in Table 4. The design effectively captured a broad spectrum of operational responses, with final moisture levels varying significantly from 7.2% to 41.2% and *a*_*w*_ values ranging from 0.57 to 0.83.

**Table 4:** Experimental Matrix of the Box–Behnken Design and Observed Responses for Papaya Seed Drying

**Run**	***T*** **(**°**C)**	***t*** **(min)**	***Q*** **(m** ^**3**^ **min** ^–**1**^ **)**	***X***_**1**_	***X***_**2**_	***X***_**3**_	***U*** (% w.b.)	***a***_***w***_
1	40	20	1.5	–1	–1	0	41.2	0.83
2	60	20	1.5	1	–1	0	13.4	0.63
3	40	60	1.5	–1	1	0	23.4	0.71
4	60	60	1.5	1	1	0	7.2	0.57
5	40	40	1.0	–1	0	–1	29.3	0.76
6	60	40	1.0	1	0	–1	12.9	0.62
7	40	40	2.0	–1	0	1	31.4	0.77
8	60	40	2.0	1	0	1	8.2	0.58
9	50	20	1.0	0	–1	–1	28.4	0.75
10	50	60	1.0	0	1	–1	16.9	0.66
11	50	20	2.0	0	–1	1	24.3	0.72
12	50	60	2.0	0	1	1	11.2	0.60
13	50	40	1.5	0	0	0	21.1	0.69
14	50	40	1.5	0	0	0	20.7	0.69
15	50	40	1.5	0	0	0	21.8	0.70

The central points of the design (runs 13, 14, and 15) exhibited minimal variation for moisture content (∼21.2%) and water activity (∼0.69), demonstrating high experimental reproducibility and low pure error. Additionally, runs 4 and 8 successfully reduced the moisture content below 10% and reached an *a*_*w*_ ≤ 0.60. According to the stability maps established by Barbosa-Cánovas *et al* ^20^, this value is recognized as the critical thermodynamic threshold below which microbial proliferation (including bacteria, yeasts, and molds) is effectively inhibited. Reaching this state is an essential prerequisite for the prolonged biochemical preservation of the matrix, theoretically preparing the seeds for future extraction of bioactive compounds, such as benzyl isothiocyanate, pending further microbiological validation.

The experimental reproducibility and process stability were assessed using the central point replicates of the Box–Behnken design. Even with the use of a heterogeneous batch of seeds, the results showed remarkable precision. The final moisture content for the three central runs was 21.1%, 20.7%, and 21.8%, yielding a mean value of 21.2% with a low standard deviation (SD) of ± 0.55%. For water activity (*a*_*w*_), values of 0.69, 0.69, and 0.70 were obtained, resulting in an SD of ± 0.0057. These low deviations provide quantitative evidence that the modified spouted bed geometry maintained a stable regime and consistent heat and mass transfer rates, effectively absorbing the physical variability of the raw material.

#### Influence of Variables on Moisture Content

The analysis of the regression coefficients for the final Moisture Content (Table 5) reveals the hierarchy of the operational effects on the drying process. The linear terms of Temperature (*X*_1_), Time (*X*_2_), and Air Flow Rate (*X*_3_) were all statistically significant (*p* < 0.05) and exhibited negative coefficients. These negative signs correctly indicate that increasing any of these independent variables acts favorably to reduce the final moisture of the seeds. Temperature (*X*_1_) emerged as the paramount factor governing the process, presenting the highest coefficient magnitude (−10.4500), which is physically consistent with the increased thermal driving force enhancing both evaporation and internal water diffusion rates.

**Table 5:** Regression Coefficients, Standard Errors, and Significance Levels for the Moisture Content Empirical Model (*R* ^2^ = 0.9923)^a^

**Factor**	**Coefficient**	**Standard Error**	***p*** **-value**
**Intercept**	**21.20**	**0.32**	**0.0002**
**Temperature** (*X*_1_)	**–10.45**	**0.20**	<**0.001**
Temperature^2^ (*X*_1_^2^)	0.09	0.14	0.6073
**Time** (*X*_2_)	**–6.08**	**0.20**	**0.0010**
Time^2^ (*X*_2_^2^)	0.04	0.14	0.8200
**Airflow** (*X*_3_)	**–1.55**	**0.20**	**0.0157**
Airflow^2^ (*X*_3_^2^)	0.46	0.14	0.0857
**Interaction** *X*_1_ *X*_2_	**2.90**	**0.28**	**0.0091**
**Interaction** *X*_1_ *X*_3_	**–1.70**	**0.28**	**0.0258**
Interaction *X*_2_ *X*_3_	–0.40	0.28	0.2873

a Significant effects (*p* < 0.05) are highlighted in bold.

The interactive effects of temperature with time (*X*_1_ *X*_2_) and temperature with air flow (*X*_1_ *X*_3_) were statistically significant. The positive coefficient observed for the *X*_1_ *X*_2_ interaction (+2.9) is of particular physical interest. This suggests that, at simultaneously high temperatures and extended drying times, the rate of moisture reduction diminishes. This antagonistic interactive behavior characterizes the onset of the falling-rate drying period, where the superficial free water has been predominantly removed and the mass transfer becomes severely limited by the internal diffusion resistance of the dense Geldart Group D particle.

The full empirical mathematical model, integrating both linear and quadratic responses, exhibited an exceptional correlation with the experimental data, yielding a determination coefficient (*R* ^2^) of 0.9923. The coded regression equation predicting the final moisture content (*U*) is expressed as follows:

1 $$U=21.20-10.45X_{1}+0.09{X_{1}}^{2}-6.08X_{2}+0.04{X_{2}}^{2}-1.55X_{3}+0.46{X_{3}}^{2}+2.90X_{1}X_{2}-1.70X_{1}X_{3}-0.40X_{2}X_{3}$$

The analysis of variance (ANOVA) detailed in Table 6 was performed to statistically validate the empirical model and quantify the contribution of each operational variable. The ANOVA results confirm the high significance of the fitted model, clearly establishing temperature (*X*_1_) as the predominant driving force in the spouted bed drying process. This is mathematically evidenced by its overwhelming sum of squares (SS = 873.620) and remarkably high Fisher *F*-statistic (*F* = 2818.13), demonstrating that thermal energy input dictates the moisture removal rate far more critically than aerodynamic parameters.

**Table 6:** Analysis of Variance (ANOVA) for the Quadratic model of Moisture Content (% w.b.)^a^

**Source of Variation**	**SS**	**df**	**MS**	***F*-value**	***p*** **-value**
**Temperature** (*X*_1_)	**873.62**	**1**	**873.62**	**2818.13**	<**0.001**
Temperature^2^ (*X*_1_^2^)	0.11	1	0.11	0.37	0.6073
**Time** (*X*_2_)	**295.25**	**1**	**295.25**	**952.40**	**0.0010**
Time^2^ (*X*_2_^2^)	0.02	1	0.02	0.07	0.8200
**Airflow** (*X*_3_)	**19.22**	**1**	**19.22**	**62.00**	**0.0157**
Airflow^2^ (*X*_3_^2^)	3.16	1	3.16	10.19	0.0857
**Interaction** *X*_1_ *X*_2_	**33.64**	**1**	**33.64**	**108.52**	**0.0091**
**Interaction** *X*_1_ *X*_3_	**11.56**	**1**	**11.56**	**37.29**	**0.0258**
Interaction *X*_2_ *X*_3_	0.64	1	0.64	2.07	0.2873
Lack of Fit	8.95	3	2.98	9.62	0.0956
Pure Error	0.62	2	0.31		
Total	1246.88	14			

a SS: sum of squares. df: degrees of freedom. MS: mean square. Bold values indicate statistically significant effects (*p* < 0.05).

Time (*X*_2_) also demonstrated a highly significant effect (*F* = 952.40), followed by airflow (*X*_3_), which, although statistically significant (*p* = 0.0157), exerted a much smaller impact on the overall variance of the final moisture content. This suggests that once the minimum spouting velocity is adequately exceeded to guarantee fluidization, further increases in airflow contribute only marginally to the mass-transfer rate.

Crucially, the Lack of Fit term was strictly nonsignificant (*p* > 0.05). This parameter evaluates the model’s failure to represent data in the experimental domain at points that are not included in the regression. A nonsignificant lack of fit, combined with an exceptionally low pure error mean square (MS = 0.310), indicates that the residual variation is merely due to natural experimental noise rather than systematic model inadequacy. Furthermore, to definitively confirm the model’s predictive robustness and rule out potential overfitting, the adjusted determination coefficient (*R*_*adj*_^2^) was evaluated. The calculated *R*_*adj*_^2^ value was remarkably high (0.9785). The minimal discrepancy between this adjusted value and the raw *R* ^2^ (0.9923) mathematically proves that the regression is not artificially inflated by nonsignificant terms. Therefore, these combined statistical indicators effectively validate eq 1 as a highly reliable predictive tool for optimization purposes within the studied operational boundaries, fulfilling the requirements for an independent statistical validation.

Verification of the quality of fit for the mathematical model and compliance with the statistical assumptions of ANOVA were performed through residual analysis (Figure 3). The normal probability plot (Figure 3a) demonstrates that the residuals align satisfactorily with the theoretical straight line without severe tail deviations, confirming the normal distribution of experimental errors. Furthermore, the plot of raw residuals versus predicted values (Figure 3b) displays a random scatter uniformly distributed around the zero axis (predominantly between −1.5 and +1.5), with no systematic patterns or funneling effects. Together, these diagnostics confirm the homoscedasticity and independence of the residuals, definitively validating the robustness of the empirical model for predictive purposes within the studied experimental domain.

**Figure 3:**
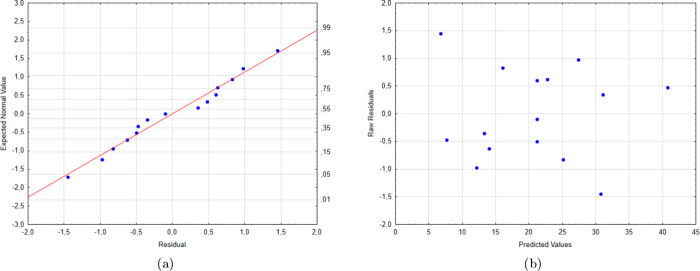
Statistical diagnostic of the moisture content model: (a) normal probability plot of residuals, indicating normality in the error distribution; (b) raw residuals versus predicted values, demonstrating homoscedasticity and absence of systematic patterns.

To better visualize the simultaneous influence and significant interaction between air temperature (*X*_1_) and processing time (*X*_2_), the response surface and its corresponding contour plot are presented in Figure 4. The three-dimensional plot illustrates the behavior of the final moisture content throughout the experimental domain with the air flow rate (*X*_3_) held constant.

**Figure 4:**
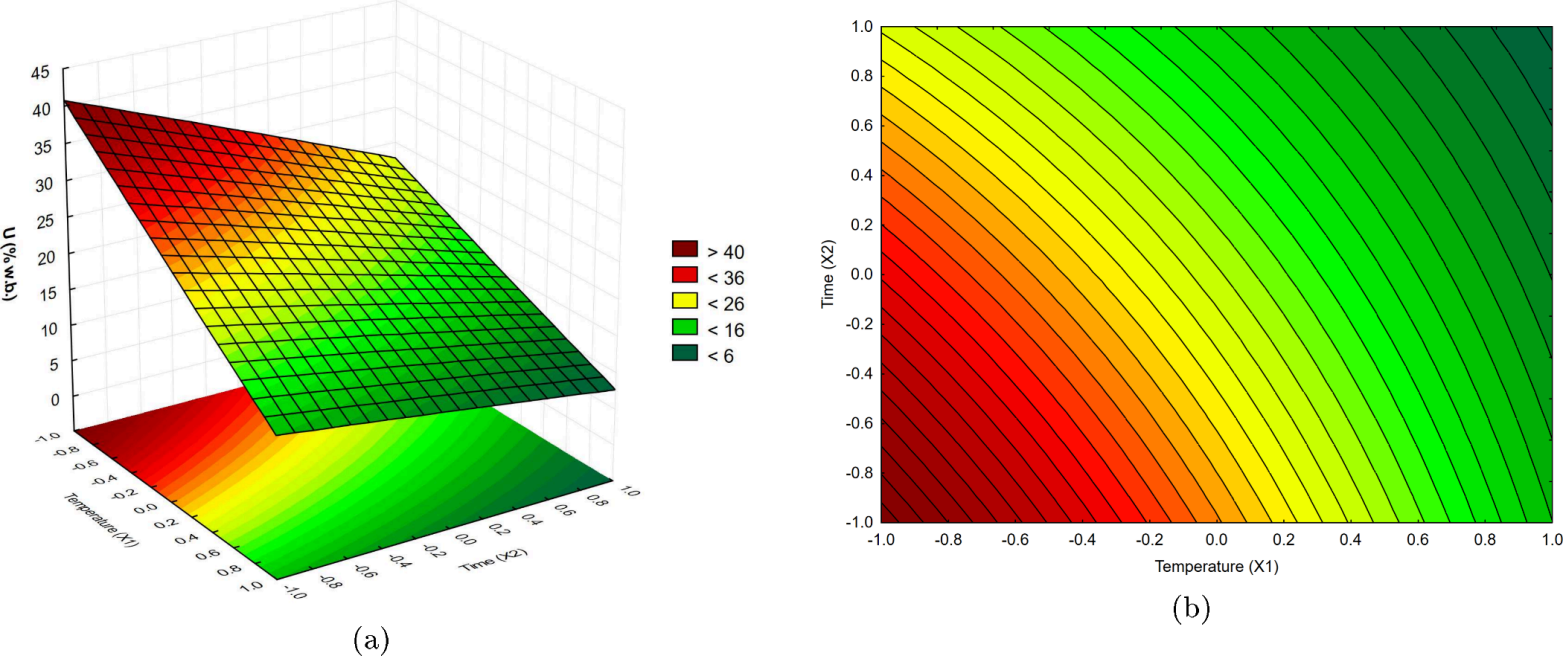
Response surface (a) and contour plot (b) for the final moisture content (*U*) as a function of temperature (*X*_1_) and processing time (*X*_2_), with airflow (*X*_3_) fixed at its central level. The plots illustrate the synergic effect of thermal energy and exposure time in overcoming internal diffusive resistance.

Analysis of the surface topography clearly illustrates the dominant role of the thermal driving force in reducing the moisture. Moreover, the slight twist in the plane visually confirms the antagonistic interaction (*X*_1_ *X*_2_ > 0) detected in the ANOVA. Specifically, at lower temperatures, extending the drying time results in a drastic and continuous moisture reduction. Conversely, at higher temperatures, the initial evaporation is so rapid that further extending the processing time yields diminishing returns, indicating that the seeds quickly transition into a diffusion-limited falling rate drying period.

The steepest descent of the surface converges toward the dark-green region in the contour plot, which represents the optimal operational zone. By operating at the maximum coded levels of both variables, the system effectively overcomes internal mass-transfer resistances, driving the final moisture content below 10% and successfully meeting the stringent preservation requirements for the biomaterial.

#### Influence of Variables on Water Activity

The regression coefficients for the *a*_*w*_ response are given in Table 7. The empirical model exhibited an excellent quality of fit to the experimental data, yielding a coefficient of determination (*R* ^2^) value of 0.9919.

**Table 7:** Regression Coefficients, Standard Errors, and Significance Levels for the Water Activity (*a*_*w*_) Empirical Model (*R* ^2^ = 0.9856)^a^

**Factor**	**Coefficient**	**Standard Error**	***p*** **-value**
**Intercept**	**0.693**	**0.003**	<**0.001**
**Temperature** (*X*_1_)	**–0.084**	**0.002**	**0.0006**
Temperature^2^ (*X*_1_^2^)	–0.004	0.003	0.2999
**Time** (*X*_2_)	**–0.049**	**0.002**	**0.0017**
Time^2^ (*X*_2_^2^)	–0.004	0.003	0.2999
**Airflow** (*X*_3_)	**-0.015**	**0.002**	**0.0180**
Airflow^2^ (*X*_3_^2^)	–0.007	0.003	0.1567
**Interaction** *X*_1_ *X*_2_	**0.015**	**0.003**	**0.0351**
**Interaction** *X*_1_ *X*_3_	**–0.013**	**0.003**	**0.0494**
Interaction *X*_2_ *X*_3_	–0.008	0.003	0.1217

a Significant effects (*p* < 0.05) are highlighted in bold.

Mirroring the behavior observed for macroscopic moisture content, the linear terms of Temperature (*X*_1_), Time (*X*_2_), and Air Flow Rate (*X*_3_) all presented statistically significant (*p* < 0.05) negative coefficients. This indicates that intensifying any of these operational parameters successfully drives the reduction of water activity. Once again, Temperature (*X*_1_) emerged as the dominant governing factor (coefficient of −0.0837). Physically, higher thermal energy input effectively breaks the intermolecular hydrogen bonds between the bound water and the complex biopolymeric matrix of the seed (such as the residual mucilage and internal cellular structures), increasing water mobility and facilitating its evaporation.

Furthermore, statistical significance was verified for two binary interactions that exhibited distinct thermodynamic behaviors. The interaction between temperature and time (*X*_1_ *X*_2_) showed a positive coefficient (+0.0150). Similar to the moisture content modeling, this slight antagonistic effect suggests that, after extended periods at elevated temperatures, the easily removable free water is depleted. Consequently, removing the remaining tightly bound monolayer and multilayer water becomes progressively more difficult, reducing the rate of the *a*_*w*_ decline.

Conversely, the interaction between Temperature and Air Flow Rate (*X*_1_ *X*_3_) exhibited a significant negative coefficient (−0.0125), corroborating a synergistic favorable effect on *a*_*w*_ reduction. This phenomenon is highly consistent with convective mass transfer principles: while the high temperature provides the necessary latent heat of vaporization to mobilize internal moisture, the high turbulent air flow continuously sweeps the evaporated water from the particle’s surface. This constant renewal of the boundary layer maximizes the vapor concentration gradient between the seed surface and the drying air, optimizing the removal of structural moisture. Notably, none of the quadratic terms demonstrated statistical significance within the established confidence level.

Based on the estimated regression coefficients, the predictive empirical eq 2 was generated for water activity (*a*_*w*_):

2 $$a_{w}=0.693-0.084X_{1}-0.004{X_{1}}^{2}-0.049X_{2}-0.004{X_{2}}^{2}-0.015X_{3}-0.007{X_{3}}^{2}+0.015X_{1}X_{2}-0.013X_{1}X_{3}-0.008X_{2}X_{3}$$

Statistical evaluation of the second-order empirical model for water activity is summarized in Table 8. Aligning with the moisture removal profile, temperature (*X*_1_) exerts the most severe influence on the thermodynamic availability of the residual water, which is reflected in its massive Fisher F-statistic (*F* = 1683.38). Exposure time (*X*_2_) also proved highly influential (*F* = 570.38), whereas airflow (*X*_3_) presented a marginal contribution, serving primarily to sustain the spouting regime rather than actively accelerating the desorption of tightly bound water.

**Table 8:** Analysis of Variance (ANOVA) for the Quadratic Model of Water Activity (*a*_*w*_)­^a^

**Source of Variation**	**SS**	**df**	**MS**	***F*-value**	***p*** **-value**
**Temperature** (*X*_1_)	**0.05611**	**1**	**0.05611**	**1683.38**	**0.0006**
Temperature^2^ (*X*_1_^2^)	0.00006	1	0.00006	1.92	0.2999
**Time** (*X*_2_)	**0.01901**	**1**	**0.01901**	**570.38**	**0.0017**
Time^2^ (*X*_2_^2^)	0.00006	1	0.00006	1.92	0.2999
**Airflow** (*X*_3_)	**0.00180**	**1**	**0.00180**	**54.00**	**0.0180**
Airflow^2^ (*X*_3_^2^)	0.00016	1	0.00016	4.92	0.1567
**Interaction** *X*_1_ *X*_2_	**0.00090**	**1**	**0.00090**	**27.00**	**0.0351**
**Interaction** *X*_1_ *X*_3_	**0.00063**	**1**	**0.00063**	**18.75**	**0.0494**
Interaction *X*_2_ *X*_3_	0.00023	1	0.00023	6.75	0.1217
Lack of Fit	0.00058	3	0.00019	5.75	0.1517
Pure Error	0.00007	2	0.00003		
Total	0.07957	14			

a SS: sum of squares. df: degrees of freedom. MS: Mean Square. Bold values indicate statistically significant effects (*p* < 0.05).

The structural integrity of the proposed equation is strongly supported by the lack-of-fit test, which yielded a nonsignificant result (*p* > 0.05). Coupled with a practically negligible pure error mean square (MS = 0.000033), this confirms that any minor deviations from the predicted curve are intrinsically linked to normal biological variability and experimental noise rather than mathematical flaws in the regression. To provide rigorous independent validation and preclude any hypothesis of data overfitting, the adjusted *R*-squared (*R*_*adj*_^2^) was computed as an additional performance metric. With a high value of 0.9774, which closely mirrors the nominal *R* ^2^ (0.9919), this parameter demonstrates that the polynomial equation accurately captures the physical phenomena without relying on extraneous or artificial terms. Ultimately, these combined indicators establish the model as a highly trustworthy framework for simulating and optimizing the stability limits of the biomaterial.

Similar to the moisture content evaluation, the residual diagnostics for the water activity model (Figure 5) confirm its statistical robustness. The normal probability plot (Figure 5a) shows a tight alignment of the residuals along the theoretical normal distribution line. Simultaneously, the scatter plot of raw residuals versus predicted values (Figure 5b) exhibits a random structureless distribution predominantly around the zero axis. These results validate the fundamental ANOVA assumptions of normality and homoscedasticity, ensuring that the model’s high predictive capacity is free from systematic bias.

**Figure 5:**
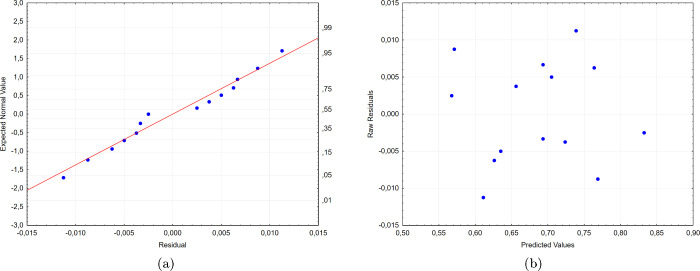
Statistical diagnostic of the water activity (*a*_*w*_) model: (a) normal probability plot of residuals; (b) raw residuals versus predicted values. The random distribution of residuals confirms the adequacy of the quadratic model for predicting the final *a*_*w*_ of papaya seeds.

The topographic behavior of the water activity across the experimental domain is visually represented in the response surface and contour plots (Figure 6). The surface analysis perfectly corroborates the ANOVA findings: the most pronounced overall reduction in *a*_*w*_ is achieved through the simultaneous increase in temperature (*X*_1_) and processing time (*X*_2_). However, the slight curvature of the plane reflects the detected antagonistic interaction (*X*_1_ *X*_2_ > 0). This visualizes the thermodynamic reality of drying biological matrices: while free water is rapidly removed, extracting the tightly bound monolayer water during the latter stages requires continuously high thermal energy but yields progressively smaller reductions in *a*_*w*_.

**Figure 6:**
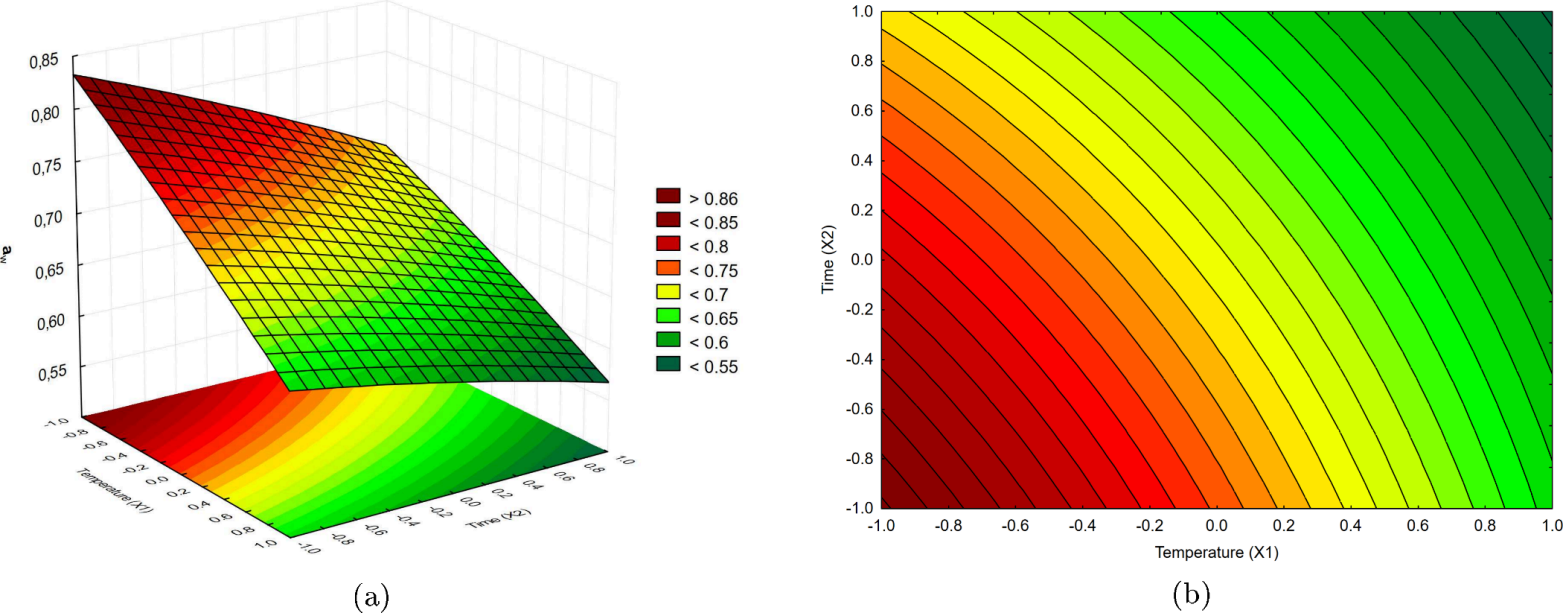
Response surface (a) and contour plot (b) for water activity (*a*_*w*_) as a function of temperature (*X*_1_) and processing time (*X*_2_), with airflow (*X*_3_) fixed at its central level. The results demonstrate the efficiency of the spouted bed in reaching the safety limit for storage (*a*_*w*_ < 0.6).

Ultimately, the optimal operational zone indicated by the dark green region in the contour plot pinpoints the exact conditions necessary to consistently achieve an *a*_*w*_ ≤ 0.60. Operating within this optimized window guarantees the long-term microbiological stability of the papaya seeds, effectively preparing the biomaterial for subsequent high-yield extraction processes.

#### Multiresponse Optimization via Desirability Profile

To determine the optimal drying conditions that simultaneously minimize the final moisture content (*U*) and water activity (*a*_*w*_) of the papaya seeds, a multiresponse optimization was performed using the desirability profile method. The desirability functions were constructed by assigning equal importance to both responses, with weights and importance factors (exponents *s* and *t*) set to 1.0. This configuration ensures a linear relationship between the experimental data and the convenience functions, providing balanced optimization.

The global desirability (*D*) was calculated based on a numerical search conducted over a grid of 50 steps for each factor within the experimental range. The results of this optimization are illustrated in Figure 7. The profile indicates that the maximum desirability (*D* = 1.0) is achieved at the highest levels of the studied variables: temperature ≈ 60 °C, drying time ≈ 60 min, and an airflow *=* 1.61 m^3^ min^–1^.

**Figure 7:**
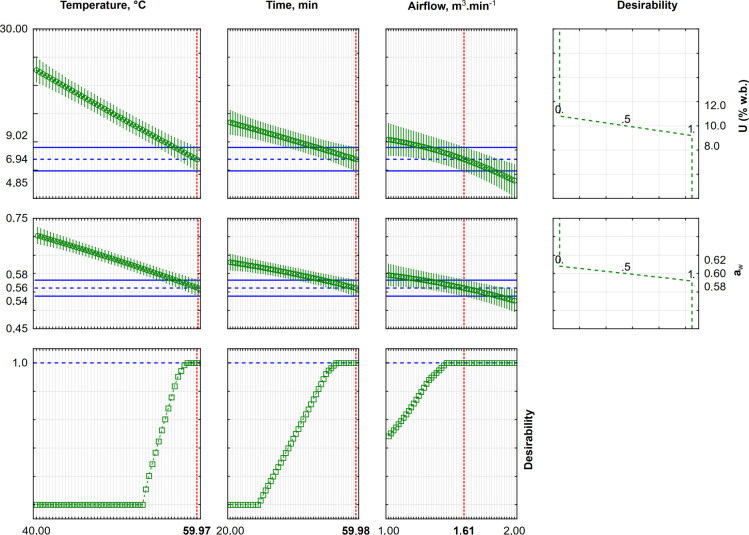
Profiles of predicted values and desirability for the multiresponse optimization of papaya seed drying. The vertical dashed lines indicate the optimal conditions.

It is worth noting that these optimized conditions show high convergence with the experimental results obtained in run 4 of the experimental design, as summarized in Table 9.

**Table 9:** Comparison between the Optimized Values Predicted by the Desirability Profile and the Experimental Results Obtained in Run 4

**Response**	**Predicted (Desirability)**	**Experimental** (Run 4)
Moisture content, *U* (% w.b.)	6.94	7.20
Water activity, *a*_*w*_	0.56	0.57

This agreement between the statistical prediction and the experimental observation validates the predictive capability of the quadratic models and confirms the efficiency of the spouted bed in reaching the safety limits for seed storage (*a*_*w*_ < 0.6) within the investigated experimental domain.

## Conclusions

This study successfully demonstrated the technical feasibility and high efficiency of using a conical-cylindrical spouted bed for drying papaya seeds (*Carica papaya* L.), seamlessly integrating fluid dynamics principles with advanced statistical optimization.

Initial physical characterization classified the fresh seeds strictly as Geldart group D particles, validating the necessity of the spouted bed to overcome conventional gas–solid contact limitations. The fluid dynamic analysis revealed that the moisture-rich sarcotesta severely dictates the initial bed behavior, inducing strong cohesive forces, pronounced hysteresis, and pressure drops characteristic of fixed-bed channeling, which were reasonably approximated by the Ergun equation. In contrast, the absence of the mucilage re-established the classical spouting regime, with the minimum spouting velocity (*u*_*ms*_) being accurately predicted by the Mathur–Gishler correlation. Furthermore, the expanded inlet nozzle design allowed for stable and highly energy-efficient fluidization at significantly lower velocities than those of standard equipment. Specifically for the drying phase, to overcome the interparticle cohesion within the heterogeneous batch, a flash drying pretreatment proved highly effective in structurally breaking down the mucilage, ensuring the stable cyclic motion required for the statistical optimization.

Regarding the drying process, the Box–Behnken design yielded robust, second-order empirical models with exceptional predictive capacity (*R* ^2^ > 0.99) for both final moisture content and water activity (*a*_*w*_). Analysis of variance (ANOVA) unequivocally identified the temperature as the predominant parameter governing mass transfer, followed closely by the processing time. The response surfaces confirmed that simultaneously high temperatures and extended drying times are required to overcome the severe internal diffusive resistance inherent to these dense biomaterials.

The most effective experimental conditions successfully drove the moisture levels below 10% (w.b.) and reduced the water activity to the critical threshold of *a*_*w*_ ≤ 0.60, a level generally associated in the literature with microbiological safety. By dynamically converting a complex, highly cohesive environmental liability into a physically stabilized biomaterial, this engineered process presents a promising pathway for the valorization of agro-industrial residues, aligning this study with the broader principles of Sustainable Development Goal (SDG) 12, offering a scalable technological foundation that can support future efforts in mitigating waste and maximizing the reuse of food byproducts.

While this study successfully established the fluid dynamic and thermodynamic boundaries for processing papaya seeds, its scope was limited to macroscopic physical stabilization. The specific impacts of thermal and aerodynamic stress on postdrying biochemical quality, such as phenolic compound retention, lipid oxidation, and extraction yields, were not quantified. Acknowledging this limitation, future interdisciplinary investigations should prioritize these biochemical metrics to fully validate the bioindustrial viability of the dried byproduct.

## Methods

### Legal and Ethical Considerations

The present study was registered in the National System for the Management of Genetic Heritage and Associated Traditional Knowledge (SisGen) under record number AD8CD49, in compliance with Brazilian Law No. 13,123/2015 regarding the access and use of components of the Brazilian genetic heritage.

### Raw Material Preparation and Characterization

Papaya fruits (*Carica papaya* L., var. Formosa) at a maturity stage corresponding to 75–100% yellow skin were acquired at a local market. The fruits were sectioned longitudinally and the seeds were manually extracted using a spoon, followed by washing under running water to remove residual pulp. The seeds were then divided into 250 g batches and immediately frozen in a domestic freezer to halt degradation. Prior to the experiments, the batches were thawed under running water at room temperature to remove surface ice, left to drain on a sieve for approximately 2 h for complete thawing, and finally dried gently with a cloth to remove external moisture.

To conduct the isolated fluid dynamic study, the sarcotesta was completely removed by manually rubbing the seeds against a Tyler no. 14 mesh sieve under running water. This process was performed in 10 min intervals, continuously separating the clean seeds to facilitate the processing of the remainder.

For the drying experiments, however, the seeds were used without a rigorous mucilage removal protocol. Instead, they were subjected only to brief sieving to remove excess pulp. During the subsequent manual handling and surface drying with a cloth, the sarcotesta of some seeds naturally ruptured, resulting in a heterogeneous batch with a visual predominance of seeds with intact mucilage, this approach reflects typical processing conditions, and its primary scientific purpose in this study was to evaluate the hydrodynamic resilience of the modified spouted bed. The presence of a heterogeneous mixture rigorously tests the equipment’s capacity to maintain stable fluidization, despite varying interparticle cohesive forces.

Physical characterization was conducted to determine the geometric parameters and density, essential for Geldart classification. The main orthogonal dimensions were measured in triplicate using a caliper, considering random sampling (*n* = 80). The density of the particles (ρ_*p*_) was determined by the liquid pycnometry method, using distilled water.

### Fluid Dynamic Study

The experiments were conducted in a spouted bed dryer (Model FBD 3.0), with a conical-cylindrical configuration (Figure 8). The column comprises a conical base with a height of 35.83 cm and an internal angle of 40°, coupled to a cylindrical section with an internal radius of 17.5 cm and a height of 34.0 cm. The drying fluid is admitted through an inlet nozzle with a radius of 4.87 cm located at the base of the cone. The system is equipped with a separation cyclone for recovery of elutriated fines.

**Figure 8:**
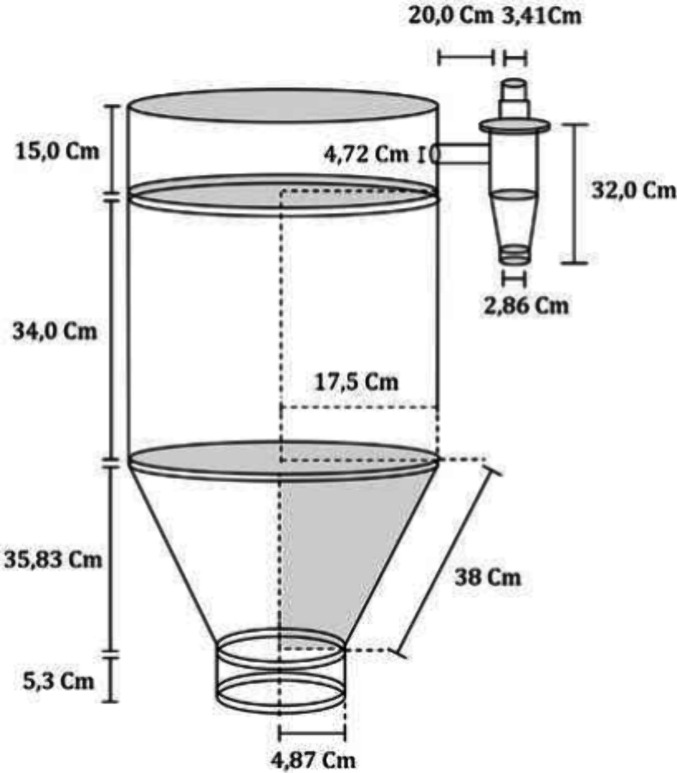
Geometric dimensions of the experimental apparatus.

It is worth noting that the geometry of the equipment used in this study features an inlet diameter (*D*_*i*_ = 9.74 cm) larger than conventional configurations reported in the literature. Recent studies demonstrate that modifications in the base and nozzle geometry drastically alter flow patterns.^16–18^

In the present work, the high *D*_*i*_/*D*_*c*_ ratio tends to reduce the initial pressure peak, facilitating the onset of fluidization for group D particles. However, this geometric configuration may hinder the direct comparison of absolute *u*_*ms*_ values with classical correlations developed for standardized geometries. Therefore, any discrepancies observed in relation to the Ergun and Mathur–Gishler models should be interpreted considering this geometric specificity, which lines up with current trends in process intensification,^17^ aiming to adapt the equipment to the physical characteristics of the biomass, rather than being limited to traditional rigid configurations.

The fluid dynamic characterization began with the measurement of the pressure drop of the empty equipment for proper correction of the experimental data, followed by loading 250 g of seeds subjected to a manual compaction protocol aimed at standardizing the height and porosity of the static bed. The pressure drop across the bed (*ΔP*) was recorded using a digital differential pressure sensor. The sensor probes were connected directly to the main air inlet tube (blower feed) and to the upper section of the cylindrical column (adjacent to the cyclone exhaust).

To ensure experimental reproducibility and adequately map the measurement system’s margin of error, all fluid dynamic mappings were executed in independent triplicate. The system behavior was mapped by constructing characteristic curves of pressure drop as a function of the superficial air velocity, operating under controlled hysteresis cycles to identify flow regimes. From the graphical analysis of the defluidization curves (representing the mean of the triplicates), the experimental minimum spouting velocities (*u*_*ms*_) were determined, which were subsequently validated by comparison with theoretical values predicted by the classical correlations of Ergun^13^ and Mathur–Gishler,^11^ respectively, considering the geometric specificities of the conical chamber and quantifying the agreement of the models by calculating the percent relative deviation.

### Experimental Design and Statistical Analysis

To evaluate the influence of the drying process operating conditions on the final characteristics of *Carica papaya* L. seeds, Response Surface Methodology (RSM) was employed. The experimental design adopted was the Box–Behnken design, chosen for its efficiency in estimating the coefficients of a quadratic model and for avoiding the simultaneous combination of all factors at their extreme levels, which could excessively degrade the biological sample. Three independent factors were selected (*k* = 3): drying air temperature (*X*_1_), process time (*X*_2_), and air flow rate (*X*_3_). Each variable was studied at three coded levels, as shown in Table 10. The seed load was kept constant at 250 ± 0.5 g.

**Table 10:** Levels of the Independent Variables

		**Coded Levels**		
**Independent Variable**	**Symbol**	**–1**	**0**	**+1**
Air temperature (°C)	*X*_1_	40	50	60
Process time (min)	*X*_2_	20	40	60
Air flow rate (m^3^ min^–1^)	*X*_3_	1.0	1.5	2.0

The design resulted in 15 experimental runs, including 3 replicates at the center point for error estimation. The execution order was randomized. The response variables analyzed were moisture content (*U*, % w.b.) and water activity (*a*_*w*_). The data were fitted to a second-order polynomial model, and statistical significance was verified by ANOVA (95% confidence level, α = 0.05), with the quality of fit evaluated by *R* ^2^. The software *Statistica 14.0* was used for data processing.
